# Complete genome sequence of soybean geminivirus A in soybean in Korea

**DOI:** 10.1128/MRA.00553-23

**Published:** 2023-09-27

**Authors:** Sangmin Bak, Mina Kwon, Seungbin Baek, Jean Geung Min, Dong Hyun Kang, Minseok Kim, Hong-Kyu Lee, Su-Heon Lee

**Affiliations:** 1 School of Applied Biosciences, Kyungpook National University, Daegu, Republic of Korea; 2 Department of Applied Biology, Kyungpook National University, Daegu, Republic of Korea; 3 Department of Plant Medicine, Kyungpook National University, Daegu, Republic of Korea; 4 Institute of Plant Medicine, Kyungpook National University, Daegu, Republic of Korea; DOE Joint Genome Institute, Berkeley, California, USA

**Keywords:** soybean, geminivirus, SGVA, complete genome

## Abstract

Soybean geminivirus A (SGVA), a member of the family Geminiviridae, was detected in a survey of early-stage soybean. The complete genome sequence of SGVA isolate Habin was determined, revealing its characteristics and similarity to Korean and Chinese isolates. This study contributes to understanding the impact of SGVA on soybean production.

## ANNOUNCEMENT

Soybean geminivirus A (SGVA), also known as soybean stay-green-associated virus, belongs to the family *Geminiviridae* (1). It is distinct from other genera within the family *Geminiviridae*, and a definitive genus designation has not been established yet (1). SGVA has been implicated as the causal agent of stay-green syndrome, with reported occurrences in China.

**Fig 1:**
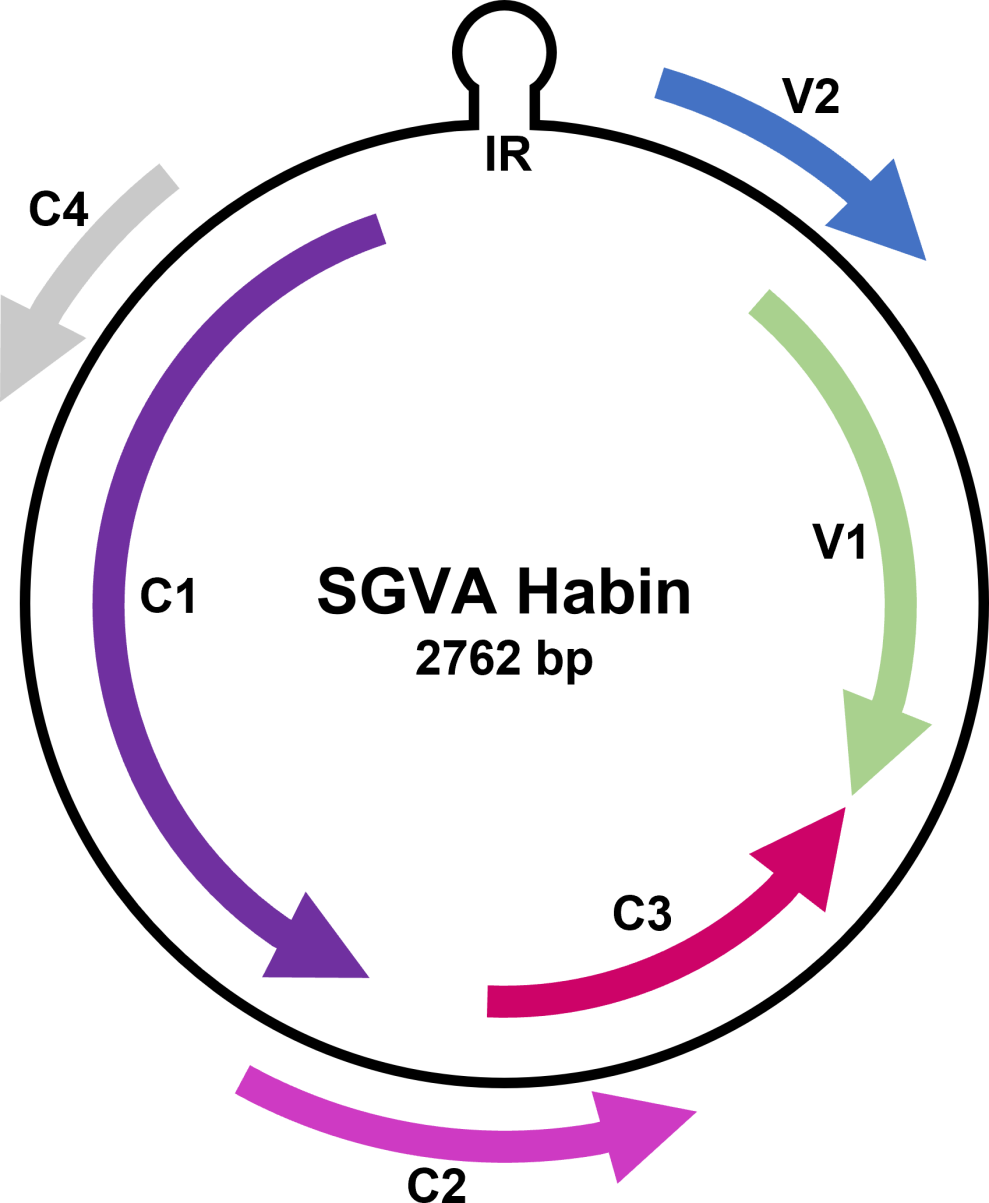
Genome schematic of soybean geminivirus A isolate Habin. The genome is a circular DNA with a length of 2,762 bp and consists of six ORFs, including two virion strand ORFs (V1 and V2) and four complementary sense ORFs (C1 to C4). The intergenic region (IR) contains the distinct nonanucleotide (5′-TAATATTAC-3′) characteristic of geminiviruses.

In June 2020, a survey was carried out at the breeding field of National Institute of Crop Science in Daegu, Korea, to investigate viral diseases in the early-stage soybeans (2). Eighty-three leaf samples exhibiting abnormal symptoms such as chlorosis, mosaic, mottle, and yellowing were collected. The samples were pooled and homogenized with liquid nitrogen. Total RNA was extracted using the Maxwell 16 LEV Plant RNA Kit (Promega, Madison, USA) and was utilized to construct single cDNA library using the TruSeq Stranded Total RNA LT Sample Prep Kit (Illumina, San Diego, USA). Subsequently, 100-bp paired-end sequencing was performed using the Illumina HiSeq 4000 platform (Macrogen, Seoul, Korea). Approximately 40 Gbp of raw data, consisting of 396 million reads, was generated, followed by the utilization of Trinity software (version trinityrnaseq_r20140717) with an *e*-value cutoff of 10^−5^ for *de novo* assembly. In the generated 119,866 contigs, three contigs each were associated with soybean mosaic virus (SMV) and peanut stunt virus (PSV), while one contig each was identified as related to soybean yellow common mosaic virus (SYCMV) and soybean yellow mottle mosaic virus (SYMMV) through NCBI BLASTn analysis. Additionally, using the same method, four contigs exhibiting a nucleotide identity of 99.4%–100.0% to the SGVA isolate King (MH428829.1) were detected. Primers were designed based on these contigs to confirm the presence of the viruses. Subsequent reverse transcription-polymerase chain reaction (RT-PCR) assay detected SGVA in 1 sample, SMV in 22 samples, SYMMV in 28 samples, and PSV, SYCMV, and SYMMV+SMV in 1 sample each. The template for determining the complete genome sequence was extracted using Exgene Plant SV (GeneAll, Seoul, Korea) from the SGVA-positive sample. Using the obtained SGVA-related contigs and reference sequences from the GenBank, four pairs of primers were designed (Table 1) to ensure 70–200 bp overlapping of amplicons, and PCR was performed accordingly. The resulting four amplicons were cloned using All in One PCR Cloning Kit (Biofact, Daejeon, Korea) and sequenced with a Phred score 20 by ABI 3730XL DNA Analyzer (Bioneer, Daejeon, Korea). The fragments were assembled using DNAMAN software (version 7.0), resulting in a circular DNA genome named isolate “Habin” with a length of 2,762 bp and a GC ratio of 42.69% (Fig. 1). It contains six predicted open reading frames (ORFs): two virion strand ORFs (V1 and V2) and four complementary sense ORFs (C1 to C4), identified by UGENE software (version 46.0) with default parameters. Additionally, a characteristic geminivirus nonanucleotide (5′-TAATATTAC-3′) identified in the intergenic region (3). In the BLASTn search, it exhibited 99.67% identity with the Korean isolate (MH428829.1) and 99.24% identity with the Chinese isolate (OM146006.1). This study will contribute knowledge to mitigate the adverse effects caused by SGVA, recently identified to induce delayed senescence in soybeans (1).

**TABLE 1:** List of primer pairs designed for the determination of the complete genome sequence of soybean geminivirus A (SGVA)^*a*^

Pair	Oligo name	Sequence (5' to 3')	Size
1	SGVA-F744	GCTACTTTGTTAAGGGATCAGTC	925
	SGVA-R1668	GAATCGGAATTTGAAACAGTGG	
2	SGVA-F1595	GGATATTGTACAAGGGGTTCG	824
	SGVA-R2418	CTGCACGAATAACAGATTCTTC	
3	SGVA-F2208	GTTAAGCGCCTTGGCGTAAG	469
	SGVA-R2676	CTCCAATGCCATACGAGTTAG	
4	SGVA-F2592	GGCTTGTATCCTAAAACGACG	1,062
	SGVA-R891	GAAACATACCCTCCATGTATG	

^*a*^ Each primer was designed using the SGVA-related contig sequences obtained through RNA high-throughput sequencing and the sequences deposited in the NCBI GenBank.

## Data Availability

The complete genome sequence of SGVA isolate Habin has been deposited at GenBank under accession number MZ736675.1. The raw data of HTS and Sanger sequencing, which were utilized to determine the complete genome sequence of SGVA, have been deposited in the Sequence Read Archive under Bio Project accession number PRJNA985228 and PRJNA999570, respectively.
